# Congenital aflatoxicosis, mal-detoxification genomics & ontogeny trigger immune-mediated Kotb disease biliary atresia variant: SANRA compliant review

**DOI:** 10.1097/MD.0000000000030368

**Published:** 2022-09-30

**Authors:** Magd A. Kotb, Ahmed Kotb, Sahar Talaat, Sherif M. Shehata, Nabil El Dessouki, Ahmed A. ElHaddad, Gamal El Tagy, Haytham Esmat, Sameh Shehata, Mohamed Hashim, Hanan A. Kotb, Hanan Zekry, Hesham M. Abd Elkader, Sherif Kaddah, Hend E. Abd El Baky, Nabil Lotfi

**Affiliations:** a Department of Pediatrics, Faculty of Medicine, Cairo University, Egypt; b Department of Pathology, Faculty of Medicine, Cairo University, Egypt; c Department of Pediatric Surgery, Faculty of Medicine, Tanta University, Egypt; d Department of Pediatric Surgery, Faculty of Medicine, Cairo University, Egypt; e Department of Pediatric Surgery, Faculty of Medicine, Alexandria University, Egypt; f Department of Rheumatology and Rehabilitation, Faculty of Medicine, Cairo University, Egypt; g Department of Pediatric Surgery, Faculty of Medicine, Ain Shams University, Egypt; h Department of Pediatrics, Faculty of Medicine, Cairo University, Egypt; i Faculty of Medicine, Cairo University, Egypt.

**Keywords:** aflatoxin induced cholangiopathy, biliary atresia, biliary atresia Kotb disease, congenital aflatoxicosis aflatoxin B1, glutathione S transferase, neutrophil elastase p53

## Abstract

Biliary atresia (BA) is the most common indication for pediatric liver transplantation. We describe The BA variant: Kotb disease. Liver tissue in the Kotb disease BA is massively damaged by congenital aflatoxicosis resulting in inflammation, adhesions, fibrosis, bile duct proliferation, scarring, cholestasis, focal syncytial giant cell transformation, and typical immune response involving infiltration by CD4+, CD8+, CD68+, CD14+, neutrophil infiltration, neutrophil elastase spill, heavy loads of aflatoxin B1, accelerated cirrhosis, disruption of p53 and GSTPi, and have null glutathione S transferase M1 (GSTM1). All their mothers are heterozygous for GSTM1. This inability to detoxify aflatoxicosis results in progressive inflammatory adhesions and obliterative cholangiopathy early in life. The typical disruption of both p53 and GSTPi causes loss of fidelity of hepatic regeneration. Hence, regeneration in Kotb disease BA typically promotes accelerated cirrhosis. The immune response in Kotb disease BA is for damage control and initiation of regeneration, yet, this friendly fire incurs massive structural collateral damage. The Kotb disease BA is about actual ongoing hepatic entrapment of aflatoxins with lack of ability of safe disposal due to child detoxification-genomics disarray.

The Kotb disease BA is a product of the interaction of persistent congenital aflatoxicosis, genetic lack of GSTM1 detoxification, ontogenically impaired activity of other hepatic detoxification, massive neutrophil-elastase, immune-induced damage, and disturbed regeneration. Ante-natal and neonatal screening for aflatoxicosis, avoiding cord milking, and stringent control of aflatoxicosis content of human, poultry and live-stock feeds might prove effective for prevention, prompt diagnosis and management based on our recent understanding of its patho-genomics.

## 1. Introduction

Biliary atresia (BA) affects the extrahepatic bile ducts in neonates and infants. The BA is associated with cholestasis and accelerated march to cirrhosis. The etiology of BA has been long unknown, yet the associations and consequences of biliary obstruction of extrahepatic bile ducts such as cholestasis, portal hypertension, cirrhosis and liver cell failure are well defined.^1^ Population based studies depicted variable incidence across countries, being almost 1:10,000 live births in Japan,^2^ 1.7–1.85:10,000 live births in Taiwan^3^ and one in 23,600 live births in Croatia.^4^

The only established line of management of BA is Kasai drainage portoenterostomy, yet almost 50% of those undergoing portoenterostomy will eventually need liver transplantation after a protracted course punctuated by attacks of cholangitis.^5^ Liver-associated damage of BA in these infants renders the native liver survival 20.3% −75.8% at 1–3 years of age, and compromise the survival at 10 years to reach 24% −52.8%.

BA has uniform cardinal features outlined in Table 1. BA is an active progressive bile duct disease, that ends in fibrosis and obliterative adhesions and that involves portahepatis more than liver tissues away from portahepatis, with unanimous immune involvement. BA has no cure and its treatment is palliative portoenterostomy.^1,23,26^

**Table 1:** Evidence-supported Features of Biliary Atresia.

	Cardinal Features	Description
1	BA is an active progressive bile duct disease	BA develops early in life ending in hurried adhesions, fibrosis and obliteration of extrahepatic bile ducts, commonly preceded with a disease free interval with possible previous intrauterine accident. The bile duct disease is always associated with insult to hepatocytes, hence relentless cholestasis.^6,7^
2	BA ends in fibrosis and obliterative adhesions	The fibrosis and adhesions end in various obliterations of bile ducts,^8^ with lack of rule in progression of disease. Seasonal variation was suspected but not definitely proven.^9–11^
3	BA inflammatory process involves portahepatis more than liver tissues away from portahepatis	There is selective increased brunt of disease damage and inflammation on cholangiocytes, and on portahepatis ^1^ with unanimous small vessel disease, ischemia, and portal vein disease.^12–17^
4	Immune involvement is unanimous	CD4+, CD8 + lymphocytes, CD68 + macrophages, CD14 + macrophages,^1,18^ and neutrophils are present in all portal tracts and in almost 70% of cases are widely distributed in the parenchyma.^12,19^ A subset of infants with BA demonstrate TH2 cytokine involvement,^20^ and other suggest a role for TH17 inflammatory pathway.^21^ Monocytes involvement in biliary atresia is recognized through Fas ligand expression on bile ductular epithelia.^22^
5	BA has no cure	There is no known cure for BA, and portoenterostomy might halt or slow the process with inconsistent effects of steroids and antibiotics. ^23,24^
6	BA treatment is portoenterostomy	Timely debulking of portahepatis is not “curative” and the “course” is plighted by attacks of cholangitis that does not comply with a predicted model. Attempts at regeneration are chaotic defying ontogeny.^5,13,25^

BA = biliary atresia, CD4+ = Cluster of differentiation 4/ T helper cells, CD8+ = Cluster of differentiation 8/ T cytotoxic cells, CD14+ = Cluster of differentiation 14/ Monocytes, CD68+ = Cluster of differentiation 68/ macrophages.

Many factors were blamed as etiologies for BA including; vascular insult, immune-mediated, genetic, structural malformation and viral infections -as reo type 3 virus, rotavirus, Epstein Barr virus and cytomegalovirus infection.^27^ Table 2 summarizes environmental factors incriminated in BA. None of these studies could explain all known BA features (outlined in Table 1). Over the past 2 decades, several studies were conducted on BA patients in-depth to formulate the underlying mechanism.^1^

**Table 2:** Environmental triggers causing biliary atresia in different species.

Environmental Factor	Species affected	Comment
Biliatresone ^28^	biliary system of larval zebrafish	Authors suggested that perinatal ingestion/exposure could be responsible for development of BA in animals or humans
Rotavirus strains^29^	Murine bile ducts	Findings were contradictory and have not succeeded in achieving an obvious differentiation between causative and accidental infection of the said virus.^29^Reproducible and convincing evidence for a causative Reoviridae infection has been lacking based on objective data from highly sensitive PCR experiments.
Cytomegalovirus ^30^	Human neonatal biliary atresia	Reported to be associated with poorer outcome of BA postportoenterostomy
Aflatoxin B1 &2 ^31^	Human neonatal biliary atresia	Aflatoxin B1 blood level was high [mean ± SD = 3.8 ± 1.73 part per billion (ppb)] in clear contrast to neonatal hepatitis where aflatoxin B1 and/or B2 were undetectable. Liver tissue of sacrificed portahepatis post Kasai portoenterostomy was also found loaded with aflatoxin B1 in all studied infants with BA.
Bacterial lipopolysaccharide ^32,33^	Human neonatal biliary atresia	Bacterial lipopolysaccharide augments hepatoxicity of aflatoxins, and causes channeling of burden of toxicity and damage to hepatocytes, through damaged sinusoidal endothelial cells and activation of coagulation system. ^33^ Bacterial lipopolysaccharide also up-regulates CD 14 + monocytes. CD 14 + when activated by binding to bacterial lipopolysaccharide result in cell killing by initiating a cascade of cytokines and nitric oxide which is a cytotoxic effector.^32,34^

CD14+ = Cluster of differentiation 14/ Monocytes.

We describe a specific BA variant: the Kotb disease BA variant. The studies of Egyptian neonates to delineate the underlying pathologic mechanism in BA proved and defined a specific etiology namely the interaction of congenital aflatoxicosis in neonates with the glutathione S-transferase M1 (GSTM1) null genotype. The pathogenesis of Kotb disease involves immune mediated damage through neutrophil elastase and CD14 + activated monocytes.^32^ Immunohistochemical staining of the portoenterostomy liver core of infants with BA, provided evidence that there is disruption of p53 and GST Pi,^35^ thus disrupting the fidelity to regeneration in BA, resulting in relentless accelerated cirrhosis. Recently we provided evidence that all studied infants with BA had unanimous loads of aflatoxin B1 in their blood and in their liver tissue.^31^ In a recent report, evidence was provided that all BA infants had difficulty in detoxifying the aflatoxins as all had null glutathione S transferase M1 (GST M1) genotype, yet all their mothers were heterozygous for GST M1.^36^ BA was thus defined as Kotb Disease.^37^

**Figure 1.**
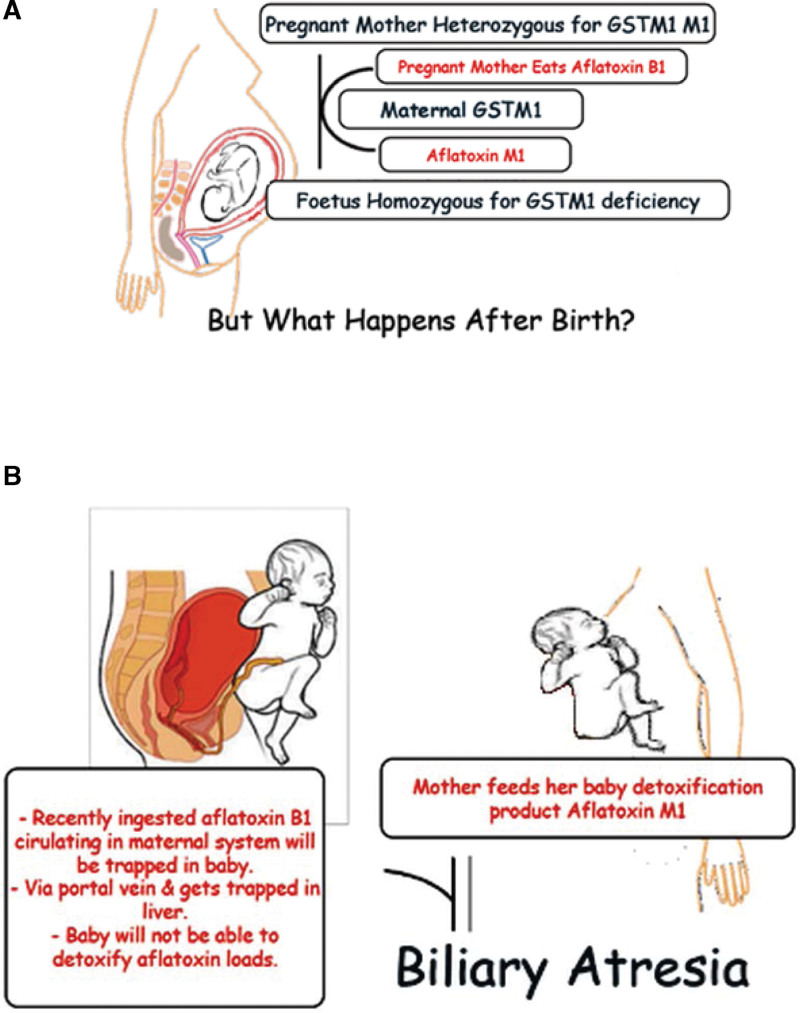
Etiology of Kotb Disease Biliary Atresia Variant in Neonates. (a) Pregnant mother heterozygous for Glutathione S-transferase M1, and her fetus is homozygous Glutathione S-transferase M1 deficient; (b) After delivery the undetoxified maternally ingested aflatoxins B1 and B2 will accumulate in the liver of the baby and initiate a massive immune response that ends in obliterative adhesions and fibrosis of extrahepatic bile ducts.

Based on the evidence published in previous extensive works and an earlier review, the pathogenesis mechanism underlying BA is depicted in Figure 1^38–40^ and Table 1.

The current review discusses the environmental triggering factors, the molecular host susceptibility factors, their interaction consequences and pathologic mechanisms implicated in Kotb disease BA variant. MEDLINE, the Cochrane Library, Scopus, and Web of Science data bases were searched for the relevant literature. Being a review article that did not involve data collected or experimental intervention in humans Ethical approval was not applicable.

## 2. Environmental factors & host susceptibility factors triggering Kotb disease BA variant

### 2.1. Toxins

Aflatoxins B1 and B2 are produced by Aspergillus flavus and rank as the number 1 carcinogens in the world.^41^ They are ubiquitous. They contaminate crops such as corn, peanuts, cottonseed, nuts, almonds, figs and spices prior to harvest or after harvest if storage conditions allow. They can also occasionally be detected in a variety of other foods and feeds. Eggs, and meat products are contaminated when the animal consumes aflatoxin-contaminated feed. However, corn, peanuts, and cottonseed remain the commodities with the highest risk of aflatoxin contamination.^42^ Aflatoxins are normally detoxified in the human body primarily by the cytochrome p450 superfamily and secondarily through glutathione conjugation.^43^ Aflatoxin M1 is a cytochrome p 450 detoxification product of aflatoxin B1. Only aflatoxin M1 (but not aflatoxin B1) is excreted in milk, thus contaminating milk products as well.^44^ Aflatoxin B1 and B2 toxicity is not limited to the genesis of hepatocellular carcinoma,^45^ they induce immune suppression,^46,47^ coagulation factor consumption and disseminated intravascular thrombosis,^48^ hemolysis,^49^ thrombosis,^50,51^ yet hepato-biliary damage remains the most consistent constant consequence.^51^

Infants with Kotb disease BA variant have abnormal loads of aflatoxin B1 in their sera, and in their liver tissue as well; in addition some infants had both aflatoxin B1 and B2 as demonstrated by the case control study performed by the Cairo University research group where the aflatoxin B1 and B2 were assessed in 2 groups of infants with cholestasis who were exclusively breastfed; a group of 24 infants with BA and a group of 17 infants with neonatal hepatitis. All enrolled infants with BA had loads of aflatoxin B1 and some had aflatoxin B2 as well as assessed by 2-dimensional thin-layer chromatography. Their aflatoxin B1 blood level was high [mean ± SD = 3.8 ± 1.73 part per billion (ppb)] in clear contrast to neonatal hepatitis where aflatoxin B1 and/or B2 were undetectable. Liver tissue of sacrificed portahepatis postKasai portoenterostomy was also found loaded with aflatoxin B1 as well [mean ± SD = 2.88 ± 0.88 ppb], and only 2 had B2 [mean ± SD = 2.58 ± 0.63 ppb], which was significantly less than their corresponding aflatoxin serum levels. In the same study the analysis of the breast milk of their nursing mothers, revealed that all maternal milk contained aflatoxin M1 and *none* contained aflatoxin B1. Aflatoxin M1 is the degradation product of aflatoxin B1. The aflatoxin M1, which was expressed in the milk of all nursing mothers of infants with BA, is also a toxin albeit milder than aflatoxin B1.^31^ All infants with BA included in this study were exclusively breastfed, and the breast milk that they nursed was analyzed and did not contain aflatoxin B1, or B2. The question of how these loads reached their livers, and the answer is as follows: aflatoxin B1 and B2 passed during labour. If so, why did they not manifest by liver disease at delivery? The answer is: BA pathogenesis starts by birth and within a few weeks obliterative fibrous adhesive obstruction of extrahepatic bile ducts sets in, followed by progressive intrahepatic cholestasis. The interaction between the toxins, genetic susceptibility, other developmental factors and immune response result in The Kotb disease BA variant.

The burden of biliary atresia follows aflatoxins in the same geographic distribution, with ethnic variability, where the Caucasians living in French Polynesia have a lower incidence of BA.^52^ Hence, genetic susceptibility is crucial in the development of BA variant Kotb disease.

### 2.2. Ontogeny and congenital glutathione s transferase deficiency in Kotb disease

Kotb disease BA is the variant that results from environment-host interaction; congenital aflatoxicosis in a host not predisposed will not develop BA, and a predisposed host not exposed to aflatoxin will not develop BA (Fig. 1).

Genotyping of GSTM1 from peripheral blood of 41 infants with BA, and from peripheral blood of all their mothers revealed that all infants had a null GSTM1 mutation concordant with homozygous deficiency, and all mothers expressed a pattern concordant with affection of only 1 allele by polymerase chain reaction (PCR).^36^

Despite the lack of studies on paternal GST M1 genotype, it would be assumed that the fathers would carry the GST M1 homozygous or heterozygous mutation, so that the off-spring with BA would inherit a GST M1 gene mutation from the father and mother. None of the mothers, however, had null mutation of GST M1, which is rational, as if she had null GST M1, she will present by a different phenotype.^53^ As the detoxification of aflatoxin is not possible during the intrauterine period of a mother with null GST M1.

Hence, infants with BA typically have a “genetic” detoxification defect; thus they cannot handle the detoxification of aflatoxin load transmitted to them before delivery. It is important to highlight that their mothers can detoxify, albeit at a slower rate, the aflatoxins all through pregnancy by their functioning GSTM1. Thus infants are mostly protected all through pregnancy from aflatoxin effects, and hence they are born normal with intact hepatic functions. The CYP 1A2 ontogenesis mostly occurs after first trimester in fetal life, up to being undetected, around 12 weeks.^54^ Hence, in case of exposure to amounts of aflatoxin during first trimester in GST M1 heterozygous mothers, inflammation of the fetal patent bile ducts might be obliterative.^55^

Again, this detoxification defect is not specific to aflatoxin, it is related to any chemical that will require GSTM1 detoxification. Thus, children with BA variant cannot handle detoxification/sulfation of other compounds as well.^56^ It might be an explanation of ursodeoxycholic acid toxicity in BA, which is reported to cause a plethora of hepatic and extrahepatic complications, and was not reported to be effective in infants with BA.^57^ Accordingly infants with BA will sustain hepatobiliary damage due to failure of detoxification of the their prenatal aflatoxin B1 loads, and the postnatal ingested aflatoxin M1 loads.^31^ (Fig. 3).

**Figure 2.**
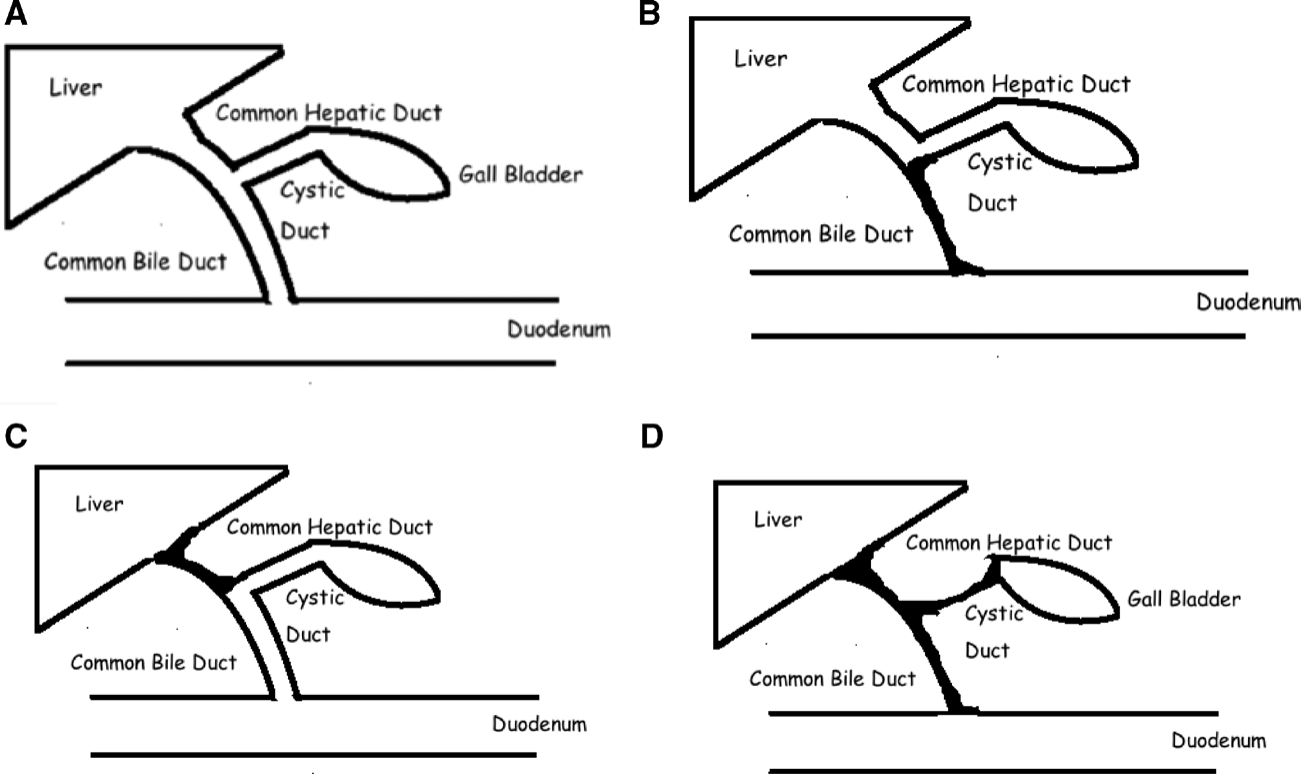
Deformed structure of extrahepatic bile ducts in biliary atresia phenotype. (a) Normal anatomy of the extrahepatic biliary system; (b) Type I biliary atresia involves obliteration of the common duct with patent gallbladder and proximal ducts; (c) Type II biliary atresia involves atresia of the hepatic duct, with cystic structures in the porta hepatis. Type II is subdivided into type IIa, where atresia is limited to the hepatic duct, with patent proximal intrahepatic, gallbladder and common bile duct, and in type IIb, the gallbladder as well as the cystic duct, common hepatic duct and common bile duct are also obliterated. (d) Type III (>90% of patients) involves atresia of the right and left hepatic ducts to the level of the porta hepatis (i.e., “complete” BA). The French designates of the above types IIa and IIb as types (2, 3) thus accordingly; there are 4 types.

**Figure 3.**
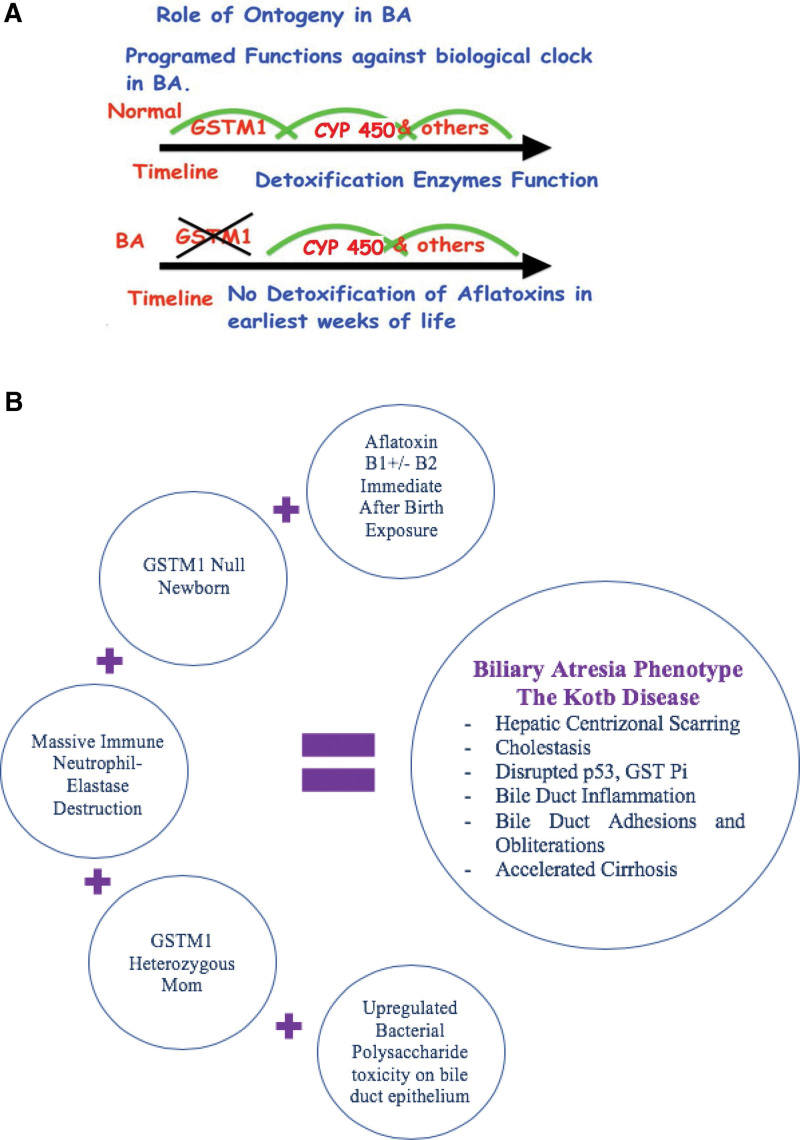
(A) Role of Ontogeny in Kotb Disease BA variant. Time line during the first, second and third months of life respectively. CYP 450 = cytochrome P450 family, others = glucuronidation. They have different expression levels and reach maturation at different ages within days, weeks, and years from birth.^53^ (B) Interaction of aflatoxins, host factors, timing and immune response in the liver in the Kotb Disease BA variant. GST = glutathione S-transferase.

### 2.3. Pathophysiology of placental detoxification of aflatoxin

In vitro studies have demonstrated that aflatoxin B1 is transferred across the human placenta. Aflatoxin B1 is metabolized by the human placenta into aflatoxicol, which is a milder toxin that is transferred by placenta to fetus and is considered detrimental to fetal health.^58^ This load of aflatoxin transfer is related to season: with higher levels in summer.^59^ CYP1A2 and 3A4, are mainly responsible for CYP-mediated AFB1 metabolism in the liver, are functional in human placenta, yet there is a natural decline in CYP 1A2 ontogenesis in fetal life, up to being undetected around 12 weeks of gestation.^54^ It is interesting however, that the cystic type of BA, that is at rare times diagnosed as early as 16 weeks of gestation,^60^ might prove to be due to exposure to aflatoxins at around the 12th week, and because of the lack of CYP1A2 activity around the same time, the fetus will develop inflammation of its developing bile ducts, incomplete adhesions, and cystic bile ducts. It is important to note that heterozygous and homozygous mutations of GSTM1 are associated with high rates of miscarriage; hence intrauterine exposure to aflatoxin in the pregnant women with heterozygous GSTM1 mutations might trigger miscarriage^53^ or cystic BA. Cystic BA might prove to be due to a different toxin interacting with another missing host detoxification enzyme, or part of the clinical phenotype due to congenital aflatoxicosis early exposure during pregnancy. Alfatoxicol was not assessed among neonates with Kotb disease. Hence, the presence of aflatoxin B1 in the porta hepatis of the affected neonates is evidence that the aflatoxin B1 that passed to the porta hepatis during umbilical cord milking was from a separated placenta that did not detoxify the whole load of aflatoxin B1, and allowed its passage to the porta hepatis of the newborn with null GSTM1.

### 2.4. Bacterial lipopolysaccharide accentuates severity of Kotb disease BA variant

Bacteria incriminated in the attacks of cholangitis in Kotb disease BA variant remain an important backstage player. Bacterial lipopolysaccharide augments the hepatoxicity of aflatoxins and causes channeling of the burden of toxicity and damage to hepatocytes, through damaged sinusoidal endothelial cells and activation of coagulation system.^51^ Bacterial lipopolysaccharide also up-regulates CD 14 + monocytes. When activated by binding to bacterial lipopolysaccharide, CD 14 + result in cell killing by initiating a cascade of cytokines and nitric oxide which is a cytotoxic effector.^32,37^

### 2.5. Ontogenic impairment of detoxification genomics early in life in Kotb disease

The neonatal period is associated with a reduction in most of detoxification pathways. The CYP1A2 is responsible for primary detoxification of aflatoxins and for 95% of demethylations in the liver. It is naturally-ontogenically-reduced during the first 2 months of life.^54^ Maturation of function of CYP1A2 occurs gradually over the earliest 1–3 months of life.^61^ Among those with BA, CYP1A2 activity is even more reduced. Detoxification during the earliest 2 months of life is very challenging for the normal off-spring.^62^ In the neonate with loads of aflatoxin, GSTM1 null deficiency, functional loss of GST Pi, and immaturity of its CYP1A2, the off-spring will struggle with the load of aflatoxin and will resort to immune-mediated removal of aflatoxin-damaged cells, and the massive immune destruction of the liver and bile ducts.^31,32,37^ (Figure 3A)

## 3. Clinico-pathological consequence of interaction of environmental & host factors in Kotb disease BA variant

Exposure to aflatoxin B1 during pregnancy, impairs fetal development and is associated with failure to thrive and anemia, ^62^ but when off-spring has null GSTM1, the exposure to aflatoxin B results in the Kotb disease BA variant.^31,37^

### 3.1. Cellular infiltration and immune system control of collateral damage in Kotb disease BA variant

Liver biopsy in BA demonstrates hepatocyte necrosis, malformed ductal plate structures; parenchymal cholate degeneration, occasional multinucleate giant cell transformation of hepatocytes,^63^ and foci of myeloid metaplasia.^64^ In Kotb disease BA variant, portal tracts are infiltrated with neutrophils,^32^ other polymorphonuclear leukocytes, monocytes, T lymphocytes and NK lymphocytes.^1,32^

### 3.2. Involvement of inflammatory molecules

The Kotb disease BA variant inflammatory response to aflatoxicosis is heralded by hepatic infiltration of CD4+, CD8 + lymphocytes, CD68 + macrophages, CD14 + macrophages,^1,32^ and neutrophils that are present in all portal tracts, and in almost 70% of cases are widely distributed in the parenchyma.^32^ This is associated with an increase in regulatory T cells.^1^

In the Kotb disease BA variant there is an abundance of antineutrophil cytoplasmic antibodies.^12^ While neutrophil activation and elastase release digest the aflatoxin-damaged bile ducts and hepatic parenchyma,^31,32^ the destruction of aflatoxin-damaged bile ducts is not limited to neutrophil elastase destruction. Other immune responses in BA include TH2 cytokine involvement,^22^ TH17 inflammatory pathway, natural killer cells (CD56(-)CD16(+) NK cells with reduced NK activity), and soluble cellular adhesion molecules^21^ have not been studied in the Kotb disease BA variant.

### 3.3. Neutrophil pivotal role in Kotb disease

Neutrophils are a unique and integral part of the innate immune system. They migrate rapidly by chemotaxis, accumulate in tissues, phagocytose and digest bacteria and fungi by their lysosymes.^65^

Neutrophils are part of the immune pathogenesis of the Kotb disease BA variant.^32^ It is peculiar that the neutrophil in action is not a simple destruction infantry battalion; it is the first step in regeneration. Neutrophil-inflicted cellular damage is a crucial step for regeneration by removal of cells that are unwanted, apoptotic, damaged or that do not demonstrate DNA fidelity.^66^ The crucial pivotal role of neutrophils in initiating the liver and bile duct damage in BA is underscored by the fact that BA has never been reported among children with Down syndrome. Children with Down syndrome are known to suffer from impaired neutrophil function.^67^

However, it is not clear if the neutrophil elastase digestion of aflatoxin-laden hepatocytes and porta-hepatis is peculiar to the Kotb Disease BA variant, and the extent of neutrophil-induced damage contribution to other BA variants.^22^ And it is unclear if the cases reported to have neutrophil infiltration in portal tracts had Kotb disease as aflatoxins were not assessed in other studies.

Liver biopsy in the Kotb disease BA variant demonstrates neutrophils in the portal tract and parenchyma as well (counts range from 2 to 12) cells per high-power field. antineutrophil elastase stain is strongly positive in all (100%) biopsies, using monoclonal antibodies against human neutrophil elastase.^32^ While neutrophils are known to stain diffusely and strongly for cationic antimicrobial protein 37 (CAP37).^68^ The latter is a neutrophil granule-derived protein that stimulates protein kinase C activity in endothelial cells. CAP37 is antimicrobial, mediates monocyte chemotaxis, and binds endotoxin.^69^ CAP37 testing in the Kotb disease BA variant remains to be studied.

When studied by immunohistochemistry neutrophil elastase is unanimous in all BA liver tissues.^32^ Also, the lack of unanimous response to steroids in controlling BA provides compelling evidence that the immune pathogenesis is not the sole determinant of outcome.^24^ Neutrophil elastase is a serine proteinase, that hydrolyzes proteins and destroys the outer membrane of E. coli, Shigella, Salmonella, and Yersinia.^70^

Moreover elastase hydrolyzes extracellular matrix and causes proteolysis of collagen-IV and elastin of the extracellular matrix,^71^ and the aflatoxin B1 bound DNA, parenchymal and nonparenchymal cells.^51^

### 3.4. Monocytes in biliary atresia

Attempts at regeneration of the biliary system start by apoptosis and/or destruction of damaged cells to be replaced by healthier cells that demonstrate cellular DNA fidelity. The involvement of monocytes in BA is recognized through Fas ligand expression on bile ductular epithelia in BA,^22^ which kills cell via a Fas/FasL-dependent pathway. Monocyte involvement through Fas ligand expression and upregulation of Fas receptor following DNA damage appears to be p53 dependent,^72^ suggesting a role in the continuous damage and obliteration of intrahepatic bile ducts after Kasai operation.^32^

Again, the disrupted p53 in BA^35^ might arrest the ontogeny-respected regeneration by arresting this monocyte-initiated pathway. Evidence for endotoxin circulation and up regulation of lipopolysaccharide endotoxin receptor CD14 + monocytes is another facet of monocyte involvement in BA.^1,37^ The CD14 + monocytes are receptors of gram-negative lipopolysaccharide endotoxins.^73^

Endotoxemia augments the tissue-damaging effect of aflatoxins up to 20 folds.^74^ The injury begins initially periportally and spreads towards midzone with time.^33^ It seems that the upregulation of CD14 + is also for initiation of apoptosis/cell killing aiming to trigger the cascade of regeneration.

### 3.5. Cellular response in BA and Kotb disease BA variant

#### 3.5.1. Bile duct proliferation.

Bile duct proliferation is constant in hepatic aflatoxicosis; hence, it is constant in BA, being a congenital aflatoxicosis, and in hepatic aflatoxicosis acquired later on in life as reported in outbreaks.^75^ Only the diameter of the bile ducts seems to affect BA outcome.^76^ Bile duct cholangiocyte proliferation is noted to follow patterns as shown in Table 3.

**Table 3:** Patterns of bile duct proliferation in cholestasis liver disease.

	Obstruction	Metaplasia	Ischemia
Feature	Elongation of tubules with respect of lumen and proliferation is confined to portal tracts	Cholangiocyte proliferation does not respect portal area, and its lumen is not well defined and is associated with inflammation and infiltration of neutrophils; moreover, these neo-formed ducts are not functionally efficient.^77^	Angiogenesis and proliferation of bile duct often in response to bile duct ischemia
Association	- Surgical bile duct ligation, - partial hepatectomy, - treatment with L-proline, - α-naphthylisothiocyanate, - taurocholate bile salts, - carbon tetrachloride (CCl4), - during acute obstructive cholestasis and in the early stages of chronic cholestatic diseases.^78^	- BA bile duct proliferation is believed to belong to the latter type. ^79^ - aflatoxin B1.^75^	- Liver transplantation, - BA. ^80^
Pathogenesis	Results from stretching and elongation of the existing bile ducts localized in the portal tracts. ^81^	It is thought of as a metaplasia that might originate from the hepatic progenitor cell rather than from the replication of preexisting ducts. Pathology is related to perinatal event. ^79,80,82^	BA is associated with reduced total hepatic blood flow. Reduced total hepatic flow is associated with progression of disease and worse outcome of BA. ^83^

BA = biliary atresia.

BA bile duct proliferation is believed to belong to the metaplasia type.^79^ Which is congruent with that described in rats subjected to aflatoxin B1.^82^

The peculiar proliferation of bile ducts and accelerated development of cirrhosis might also be related to the disruption of p53 by aflatoxin.^35^ The p53 is a cellular “replication fidelity” sentinel. Aflatoxin B1 is notorious for inducing p53 mutations that herald carcinogenicity.^84^ In BA, p53 was studied by immunohistochemical staining in portoenterostomy cores of liver tissue. All 32 studied biopsies demonstrated defective staining of p53 and glutathione S-transferase Pi.^35^

Bile duct proliferation in BA is related to obstruction, aflatoxin-induced metaplasia and ischemia.^79,80^ BA is associated with reduced total hepatic blood flow. Reduced total hepatic flow is associated with progression of disease and worse outcomes of BA.^83^

#### 3.5.2. Fibrosis.

Histologically, extensive fibrosis is present in almost all liver biopsies in of BA. Almost all patients have fibrosis of varying severity that promptly marches to cirrhosis. It is noted that pre- portoenterostomy fibrosis progresses to biliary cirrhosis postoperatively. However, in some patients a reduction in the degree of fibrosis has been reported. Hence, it was concluded that hepatic fibrosis is a dynamic process with no correlation between the clinical outcome and the severity of hepatic fibrosis score. The severity degree of fibrosis at the time of Kasai portoenterostomy seems to be related to poorer outcomes.^1,32^ (Fig. 2).

Fibrosis is expressed clinically as firmness of the liver, with rapid march to development of cirrhosis, portal hypertension, splenomegaly, ascites, and varices. ^1^ The aflatoxcosis, neutrophil elastase-induced damage and upregulation of CD 14 + contribute to the severity and extent of fibrosis in the Kotb disease BA variant.^1,32,37^

#### 3.5.3. Structural deformation.

Aflatoxin-induced hepatobiliary damage results in scarring, stenosis, fibrosis and adhesions of the lumen of the extrahepatic bile ducts.^37^ Classification of BA into 3 distinct types is based on scarring; I,II and III. Most often, the BA is complete (in 73%).^5,13,24^ (Fig. 2 and 3B). The brunt of damage is not equal or evenly distributed across the liver. Stellate cells increase at the site of acute liver injury. This might be the result of the uneven distribution of the stellate cells of Ito that secrete fibrin.^85^ Again aflatoxins have a predilection for liver damage.^75^ Hence, uniformity is not expected.

#### 3.5.4. Regeneration infidelity & cirrhosis.

BA is not a self-limiting condition despite fulfilling the neutrophil-inflicted cellular damage step, T cells, B cells, natural killer cells and monocytes involvement and healing by regeneration results in cirrhosis. Cellular p53 and GST govern the “ontogeny” respected regeneration. When governed by intact p53 and GST regeneration that results after the killing of damaged cells should respect ontogeny and should respect lobular architecture structure. However, regeneration in BA involves fibrosis, abnormal structure and accelerated cirrhosis.^1^

The only trial that investigated p53 in BA demonstrated a unanimous disruption of p53 in BA.^35^ p53 is responsible for DNA fidelity at cellular replication and regeneration, while GST, which is responsible for detoxification of a wide array of substances that affect cellular replication and DNA fidelity as well.^54,86^

In view of the strong obligate involvement of neutrophil elastase in BA, it is expected that cellular debris is removed and BA would be a self-limiting disease; instead regeneration in BA results in variable degrees of fibrosis and cirrhosis.^1^

## 4. Challenges of Kotb disease BA variant presentations

### 4.1. BA in identical twins

Reports of concordant and discordant BA among twins exist.^87^ While none tested the twins for aflatoxins or GST M1, we are inclined to interpret the random occurrence of BA among twins to be related to the amount of placental insufficiency that occurs with the placental separation^88^ and subsequent milking of the cord of this placenta that has incompletely or insufficiently detoxified the aflatoxin burden during delivery. We postulate that, the twin with null GST M1 who underwent milking of cord from a placenta that had completely detoxified the aflatoxin load did not develop the BA variant, whereas the twin with null GST M1 who underwent milking of cord from a separated placenta and did not sufficiently detoxify the aflatoxin load will develop the BA variant.

### 4.2. Intrauterine prenatal BA

Intact placental detoxification allows the breakdown and detoxification of aflatoxin. The detoxification ability is not unanimous during pregnancy, and by the end of the first trimester the placental detoxification is compromised as the ontogeny of detoxification enzymes is related to chronological events of pregnancy.^89^ Hence, exposure to aflatoxins in the maternal diet during this period might result in inflammation, adhesions and obliteration of extrahepatic bile ducts. More studies are needed to verify the amount of aflatoxin in the hepatic portal area tissue of the BA variants presenting prenatally.

## 5. Is Kotb disease BA phenotype potentially preventable?

Yes. The Kotb disease variant proved to be due to congenital aflatoxicosis in GSTM1 null neonates, resulting in massive inflammation induced by neutrophils, adhesions, and obliteration followed by fibrosis of extrahepatic biliary radicals, accelerated cirrhosis and portal hypertension. Future research is needed to study: (a) efficacy, sensitivity, specificity and cost-effectiveness of perinatal screening of pregnant women about giving birth for aflatoxins using rapid detection techniques,^90^ and abandoning milking of the cord during delivery of those who test positive for aflatoxins would prevent biliary atresia variant in the off-spring, (b) efficacy, sensitivity, specificity, and cost-effectiveness of neonatal screening for aflatoxins, and whoever tests positive for genotype of GST M1 to allow early prompt neonatal diagnosis of congenital aflatoxicosis, (c) effectiveness of strict aflatoxin-free diet on the offsprings of mothers who tested positive for aflatoxins and early portoenterostomy await future studies, (d) effectiveness of prompt exchange transfusion among the early detected cases to decrease load of aflatoxins, and (e) the possibility of gene therapy for GST M1 and stem cell transplantation that might offer affected child another means of aflatoxin- chelation, prior to cirrhosis and massive immune-aflatoxin damage. Effect of delayed umbilical cord clamping on incidence of BA remains to be studied as well.

### 5.1. Review of other BA phenotypes resulting from possible toxin & host susceptibility factors interaction model

BA appears to be a phenotype that results from diverse etiologies. Other models include:

I- Among larval zebrafish researchers have reported that *biliatresone*, which is an isoflavonoid, caused selective destruction of the extrahepatic, but not intrahepatic, biliary system. The authors suggested that perinatal ingestion/exposure could be responsible for the development of BA in animals. Biliatrsone has never been reported to cause BA in humans.^28^

II- GPC1, ADD3 and ARF6 are susceptibility genes primarily studied in zebrafish embryos, that were exposed to the toxin named biliatresone. These genes are related to organogenesis.^91^

Studies reporting on the ARF6 gene in humans have many uncertainties and are not unanimous among the infants with biliary atresia. It was studied among 61 BA and 1907 controls. However, the authors believe that, as previous studies conducted in patients with BA demonstrated a high likelihood of multiple susceptibility loci and the environment in disease pathogenesis, they suspected that future models, which cannot demonstrate such additive effects, are likely to maintain a supportive role by placing the larger burden of proof provision on the human evidence from diseased subjects“.^91^

GPC1 was also studied in 61 children with BA and 5088 controls. The region at 2q27.3 was found to be heterozygously deleted in 6 patients (9.84%) and 4 controls (0.08%) (*P* = 4.4 × 10 − 10).^92^ We believe that future studies may expose other susceptibility genes that might explain individual case associations and/or variations.

III- Murine bile ducts were also proven to be affected by rotavirus strains.^29^ Other findings were contradictory and did not succeed in achieving an obvious differentiation between causative and accidental infection of the rotavirus. Reproducible and convincing evidence for a causative Reoviridae infection has been lacking based on objective data from highly sensitive PCR experiments.^27,93,94^

IV. In addition, the cytomegalovirus BA variant was reported to be associated with poorer outcomes of BA postportoenterostomy.^30^ It is not clear however if CMV complicates BA, or contributes to BA pathogenesis.

V. Several other variants with associated congenital malformations (such as laterality defects or congenital heart, urinary or gastrointestinal diseases), which are generally grouped together as biliary atresia-splenic malformation syndrome.^95^

## 6. Conclusion and future perspectives

Future research is needed to define other etiologies responsible for the BA phenotype. Validation and reproducibility studies are required to define the frequency of the Kotb disease BA variant resulting from aflatoxicosis interaction in the GST M1 null offspring across nations and ethnicities. Kotb disease BA is a variant comprising an obstructive cholangiopathy of neonates and infants suffering from glutathione S-transferase M1 deficiency when exposed to intrauterine aflatoxins B1 and B2. Molecular studies have consistently revealed that their mothers are heterozygous for GSTM1. BA is a disease that results from host factor-environment interactions. Until safe and effective chelation therapy is introduced as a management protocol in BA, prevention seems to be the first line of management by strict watchful implementation of maximum allowable aflatoxin content in humans, poultry, and bovine foods. The Kotb disease BA is potentially preventable.

## Author contributions

All authors searched medical literature, databases, conceptualized, prepared this review and reviewed the final manuscript.

## Acknowledgments

We acknowledge late Professor Ahmed Kotb, cofounder of Pediatric Hepatology Subspecialty in Department of Pediatrics, Cairo University, Egypt.
